# Non random distribution of genomic features in breakpoint regions involved in chronic myeloid leukemia cases with variant t(9;22) or additional chromosomal rearrangements

**DOI:** 10.1186/1476-4598-9-120

**Published:** 2010-05-25

**Authors:** Francesco Albano, Luisa Anelli, Antonella Zagaria, Nicoletta Coccaro, Paola Casieri, Antonella Russo Rossi, Laura Vicari, Vincenzo Liso, Mariano Rocchi, Giorgina Specchia

**Affiliations:** 1 Hematology, University of Bari, 70124, Bari, Italy; 2 Service of Medical Genetics, Cardarelli Hospital, via Cardarelli 9, 80131 Naples, Italy; 3 Department of Genetics and Microbiology, University of Bari, 70126 Bari, Italy

## Abstract

**Background:**

The t(9;22)(q34;q11), generating the Philadelphia (Ph) chromosome, is found in more than 90% of patients with chronic myeloid leukemia (CML). As a result of the translocation, the 3' portion of the *ABL1 *oncogene is transposed from 9q34 to the 5' portion of the *BCR *gene on chromosome 22 to form the *BCR*/*ABL1 *fusion gene. At diagnosis, in 5-10% of CML patients the Ph chromosome is derived from variant translocations other than the standard t(9;22).

**Results:**

We report a molecular cytogenetic study of 452 consecutive CML patients at diagnosis, that revealed 50 cases identifying three main subgroups: i) cases with variant chromosomal rearrangements other than the classic t(9;22)(q34;q11) (9.5%); ii) cases with cryptic insertions of *ABL1 *into *BCR*, or vice versa (1.3%); iii) cases bearing additional chromosomal rearrangements concomitant to the t(9;22) (1.1%). For each cytogenetic group, the mechanism at the basis of the rearrangement is discussed.

All breakpoints on other chromosomes involved in variant t(9;22) and in additional rearrangements have been characterized for the first time by Fluorescence In Situ Hybridization (FISH) experiments and bioinformatic analyses. This study revealed a high content of *Alu *repeats, genes density, GC frequency, and miRNAs in the great majority of the analyzed breakpoints.

**Conclusions:**

Taken together with literature data about CML with variant t(9;22), our findings identified several new cytogenetic breakpoints as hotspots for recombination, demonstrating that the involvement of chromosomes other than 9 and 22 is not a random event but could depend on specific genomic features. The presence of several genes and/or miRNAs at the identified breakpoints suggests their potential involvement in the CML pathogenesis.

## Background

Chronic myeloid leukemia (CML) is characterized by the constitutive expression of the *5'BCR/3'ABL1 *fusion gene resulting from the t(9;22)(q34;q11); this translocation is evident in more than 90% of patients and produces the Philadelphia chromosome (Ph)[1].

In 5-10% of CML patients, the *5'BCR/3'ABL1 *fusion gene arises from complex variant rearrangements which may involve one or more chromosomes in addition to 9 and 22 [2,3]. In some variant t(9;22) cases, additional material is transferred onto the Ph chromosome, resulting in a "masked" Ph whereas other CML patients show a classic Ph and an atypical der(9) chromosome as a consequence of a rearrangement between the der(9)t(9;22) and another chromosome [4,5]. Serial translocations or a single simultaneous event are alternative hypotheses proposed to justify the occurrence of these complex rearrangements [6].

In a subset of CML patients, cryptic rearrangements have been postulated to induce the chimeric gene formation, such as a nonreciprocal insertion between chromosomes 9 and 22 or two sequential translocations restoring the partner chromosomes morphology [7-11].

Microdeletions on the der(9) chromosome next to the t(9;22) breakpoint have been described in patients with classic and variant Ph translocations, and appear to be a valuable prognostic factor [12-17]. Recently, the frequency of such deletions has been investigated in the subgroup of CML patients with a masked Ph chromosome [18]. Additional genomic deletions on the third derivative chromosome have also been described in CML cases with variant translocations [19,20].

To our knowledge, an accurate breakpoints identification and bioinformatic analysis of other chromosomes involved in variant t(9;22) or in concomitant chromosomal rearrangements apart from the t(9;22) has never been performed in CML.

In this paper, a detailed molecular cytogenetic characterization of 50 (11.1%) out of 452 chronic phase (CP) CML patients was carried out to define the precise breakpoints on chromosomes other than 9 and 22. Bioinformatic analysis of breakpoint regions was performed to investigate the presence of repeated elements (*Alu*, LINE), GC content, Segmental Duplications (SDs), miRNAs, and known genes. Our findings, taken together with a review of literature data, allowed us to identify new cytogenetic hotspots in CML cases with variant t(9;22).

## Methods

### Patients

The study included 452 CP-CML patients. All of them were newly diagnosed at our hospital between 1990 and 2009. As a consequence of the long time span for sample accrual, several therapeutic regimens (hydroxyurea, interferon-α, imatinib, nilotinib, and dasatinib) were adopted.

Out of the 452 CP-CML cases, 50 showed variant t(9;22) or additional chromosomal rearrangements, 9 of which have been characterized in previous reports by our group [19-23].

### Conventional cytogenetics

Conventional cytogenetic analysis of a 24-48 hour culture was performed at diagnosis of CML on bone marrow cells by standard techniques and evaluated by Giemsa-Trypsin-Giemsa (GTG) banding at about the 400-band level according to the ISCN [24]. At least 25 metaphases were analyzed for each case.

### Identification of cytogenetic hotspots

To identify new cytogenetic hotspots, an estimate of the Haploid Autosomal Length (HAL) of the bands involved in variant t(9;22) cases was performed [25,26]. We calculated the number of breaks expected (E) in any band, given the null hypothesis of a random distribution of all breaks across the genome. Reviewing large series of CML patients with variant t(9;22) we assessed the number of breaks observed (O) in each band and divided this value by the expected (E) value to determine an O/E ratio. An O/E ratio >1 identified new cytogenetic hotspots.

### FISH analysis

FISH analysis was performed on bone marrow samples of all CP-CML patients at diagnosis using "home-brew" FISH probes specific for *ABL1 *and *BCR *genes, validated in previous papers [13,16,27]. Breakpoints characterization and deletions size definition were carried out with additional bacterial artificial chromosome (BAC) and Phage P1-derived artificial chromosome (PAC) probes. All clones were selected according to the University of California Santa Cruz (UCSC http://genome.ucsc.edu/index.html; March 2006 release) database [28]; the mapping of each clone was first tested on normal human metaphases. Chromosome preparations were hybridized in situ with probes labeled with biotin by nick translation [29]. Briefly, 500 ng of labeled probe were used for FISH experiments; hybridization was performed at 37°C in 2× standard saline citrate (SSC), 50% (vol/vol) formamide, 10% (wt/vol) dextran sulphate, 5 μg COT1 DNA (Bethesda Research Laboratories, Gaithersburg, MD), and 3 μg sonicated salmon sperm DNA in a volume of 10 μL. Post-hybridization washing was done at 60°C 0.1× SSC. Biotin-labeled DNA was detected with Cy3-conjugated avidin. In cohybridization experiments, other probes were directly labeled with fluorescein. Chromosomes were identified by 4',6-diamidino-2-phenylindole (DAPI) staining. Digital images were obtained using a Leica DMRXA epifluorescence microscope equipped with a cooled CCD camera (Princeton Instruments, Boston, MA). Cy3 (red; New England Nuclear, NJ), fluorescein (green; NEN Life Science Products, Boston, MA), and DAPI (blue) fluorescence signals, which were detected using specific filters, were recorded separately as gray-scale images. Pseudocoloring and merging of images were performed with Adobe Photoshop software.

### Bioinformatic analysis

Breakpoint regions on other chromosomes involved in variant t(9;22) and additional rearrangements were included in 250 Kb size intervals, according to the resolution limit of the BAC clones used for breakpoints definition. Each interval was checked for the presence of interspersed repeats classes (*Alu *and LINE repeats), SDs, GC content, and gene density. The UCSC Table Browser [28] was queried for summary analysis about the items belonging to the tracks "RepeatMasker", "Segmental Dups", "GC Percent", and "RefSeq Genes". For SDs and RefSeq gene analysis, both "Item count" and "Item Bases" values were considered, to assess their number and the bases percentage involved in SDs or coding sequences, respectively. For each genomic feature, the obtained value was normalized to the mean value for the examined chromosome. For example, in case 1, the breakpoint mapped in 1q32.1 (chr1:203,949,120-204,199,120) showed an *Alu *frequency of 13.47%. As the mean *Alu *content inside chromosome 1 is estimated to be 11.9%, the normalized value will be 1.13 (i.e. 13.47/11.90). Therefore, greater or lesser values than 1 correspond to regions with a richer or poorer content of a specific genomic feature than those observed along the entire chromosome.

In view of the known low miRNAs density in the human genome, regions spanning 2 Mb proximally and distally to breakpoints were investigated by querying the UCSC database at the track "sno/miRNA". For each chromosome the expected miRNA density within a 4 Mb interval was established according to the following formula: number of miRNA along the entire chromosome/size in bp of the chromosome × 4000000 bp. The identification of the predicted miRNAs target genes was performed by querying the miRGen database http://www.diana.pcbi.upenn.edu/cgi-bin/miRGen/v3/Targets.cgi. Intersection data from the three widely used target prediction programs (miRanda, PicTar, TargetScan) were considered. The definition of target genes function as oncogenes or tumor suppressor genes (TSGs) was made according to the National Center for Biotechnology Information (NCBI, http://www.ncbi.nlm.nih.gov/gene/) database.

## Results

### FISH data

Cytogenetic analysis and FISH experiments with specific BAC/PAC probes for the *ABL1 *and *BCR *genes allowed us to detect 50 (11.1%) out of 452 cases, that identify 3 main subgroups of CML patients showing variant t(9;22) rearrangements, the occurrence of cryptic insertions of the *ABL1 *in the *BCR *region (or vice versa), and the presence of additional chromosomal abnormalities, respectively (Table 1).

**Table 1:** Cytogenetic groups identified in a large series of CML patients at diagnosis

*Cases*	*Chromosomal rearrangements*	*Ph chromosome*	*Deletions*	*5'BCR/3'ABL* *location*	*5'ABL/3'BCR location*
**Variant t(9;22)**					
#1	t(1;9;22)(q32.1;q34;q11)	Ph^+^	del(1)(q32), del(9)(q34), del(22)(q11)	Ph	*5'ABL *deleted, *3'BCR *on der(1)
#2	t(1;9;22)(p34.2;q34;q11)	Ph^+^	-	Ph	*5'ABL *on der(9), *3'BCR *on der(1)
#3	der(9)t(9;22)(q34;q11), der(1)ins(1;22) (p36;q11q13), der(22)t(9;22)	Ph^+^	-	Ph	*5'ABL *on der(9), *3'BCR *on der(1)
#4	t(2;9;22)(q37.1;q34;q11)	Ph^+^	-	Ph	*5'ABL *on der(9), *3'BCR *on der(2)
#5	t(3;9;22)(p21.31;q34;q11)	Ph^+^	del(22)(q11)	Ph	*5'ABL *on der(9), *3'BCR *on der(3)
#6	t(3;9;22)(p21.31;q34;q11)	Ph^+^	-	Ph	*5'ABL *on der(9), *3'BCR *on der(3)
#7	t(3;9;22)(p21.31;q34;q11)	Ph^+^	-	Ph	*5'ABL *on der(9), *3'BCR *on der(3)
#8	t(4;9;22)(p16.3;q34;q11)	Ph^+^	del(9)(q34)	Ph	*5'ABL *on der(4), *3'BCR *on der(4)
#9	t(4;9;22)(p16;q34;q11)	Ph^+^	del(9)(q34), del(22)(q11)	Ph	*5'ABL *on der(4), *3'BCR *deleted
#10	t(4;9;22)(q12;q34;q11)	Ph^+^	del(4)(q12), del(22)(q11)	Ph	*5'ABL *deleted, *3'BCR *on der(4)
#11	t(4;9;22)(p16.2;q34;q11)	Ph^+^	del(4)(p16), del(9)(q34), del(22)(q11)	Ph	*5'ABL *deleted, *3'BCR *deleted
#12	t(6;9;22)(p12.3;q34;q11)	Ph^+^	del(6)(p12), del(9)(q34)	Ph	*5'ABL *deleted, *3'BCR *on der(6)
#13	t(6;9;22)(p21.31;q34;q11)	Ph^+^	del(6)(p21p21), del(9)(q34q34), del(22)(q11q11)	Ph	*5'ABL *deleted, *3'BCR *on der(6)
#14	t(6;9;22)(q14.1;q34;q11)	Ph^+^(×2)	-	Ph	*5'ABL *on der(9), *3'BCR *on der(6)
#15	t(7;9;22)(p14.3;q34;q11)	Ph^+^	del(7)(p14), del(9)(q34), del(22)(q11)	Ph	*5'ABL *deleted, *3'BCR *deleted
#16	t(7;9;22)(p22;q34;q11)	Ph^+^	-	Ph	*5'ABL *on der(9), *3'BCR *on der(7)
#17	der(9)t(9;22)(q34;q11), der(9)ins(9;9) (q34;q22q34)	Ph^+^	-	Ph	*5'ABL *on der(9), *3'BCR *on der(9)
#18	t(9;22;9;14)(q34;q11;p12;q11)	Ph^+^	**-**	Ph	*5'ABL *on der(9), *3'BCR *on der(9)
#19	t(9;11;22)(q34;q13.1;q11)	Ph^+^	del(9)(q34), del(11)(q13), del(22)(q11)	Ph	*5'ABL *deleted, *3'BCR *deleted
#20	t(9;11;22)(q34;q21;q11)	Ph^+^	-	Ph	*5'ABL *on der(9), *3'BCR *on der(11)
#21	t(9;12;22)(q34;q23.3;q11)	Ph^+^	-	Ph	*5'ABL *on der(9), *3'BCR *on der(12)
#22	t(9;12;22)(q34;q13.2;q11)	Ph^+^	**-**	Ph	*5'ABL *on der(9), *3'BCR *on der(9)
#23	t(9;12;22)(q34;q24.31;q11)	Ph^+^	del(9)(q34), del(12)(q24), del(22)(q11)	Ph	*5'ABL *deleted, *3'BCR *on der(12)
#24	t(9;12;22)(q34;q24.21;q11)	Ph^+^	-	Ph	*5'ABL *on der(9), *3'BCR *on der(12)
#25	t(9;13;22)(q34;q14.12;q11)	Ph^+^	del(9)(q33q34), del(13)(q14), del(22)(q11)	Ph	*5'ABL *on der(13), *3'BCR *deleted
#26	der(9)t(9;22)(q34;q11), der(14)ins(14;9)(q32;q34q34), der(22)t(9;22)	Ph^+^	del(9)(q34), del(22)(q11)	Ph	*5'ABL *on der(14), *3'BCR *on der(14)
#27	t(9;14;15;22)(q34;q24.2;?;q11)	Ph^+^	-	Ph	*5'ABL *on der(9), *3'BCR *on der(14)
#28	t(9;15;22)(q34;q24.3;q11)	Ph^+^	-	Ph	*5'ABL *on der(9), *3'BCR *on der(15)
#29	t(9;16;22) (q34;p11.2;q11)	Ph^+^	-	Ph	*5'ABL *on der(9), *3'BCR *on der(16)
#30	t(9;16;17;22)(q34;q24.3;p13.1;q11)	Ph^+^	-	Ph	*5'ABL *on der(9), *3'BCR *on der(17)
#31	t(9;17;22)(q34;q21.2;q11)	Ph^+^	-	Ph	*5'ABL *on der(9), *3'BCR *on der(9)
#32	t(9;17;22) (q34;q25.3;q11)	Ph^+^	-	Ph	*5'ABL *on der(9), *3'BCR *on der(17)
#33	t(9;17;22) (q34;p13.3;q11)	Ph^+^	-	Ph	*5'ABL *on der(9), *3'BCR *on der(17)
#34	t(9;19;22)(q34;q13.32;q11)	Ph^+^	-	Ph	*5'ABL *on der(9), *3'BCR *on der(19)
#35	t(9;21;22)(q34;q22.13;q11)	Ph^+^	del(9)(q34), del(21)(q22), del(22)(q11)	Ph	*5'ABL *deleted, *3'BCR *deleted
#36	t(9;X;22)(q34;q13.1;q11)	Ph^+^	del(9)(q33q34)	Ph	*5'ABL *deleted, *3'BCR *on der(X)
#37	t(7;9;22)(q22.2;q34;q11)	masked Ph	-	der(7)	*5'ABL *on der(9), *3'BCR *on der(9)
#38	t(8;9;22)(p12;q34;q11)	masked Ph	-	der(8)	*5'ABL *on der(9), *3'BCR *on der(9)
#39	t(9;10;22)(q34;p11.22;q11)	masked Ph	del(9)(q34), del(22)(q11)	der(10)	*5'ABL *deleted, *3'BCR *deleted
#40	t(9;15;22)(q34;q24.1;q11)	masked Ph	-	der(22)	*5'ABL *on der(9), *3'BCR *on der(9)
#41	t(6;9;12;22)(p22.1;q34;q13.13;q11)	masked Ph	del(9)(q34)	der(22)	*5'ABL *on der(9), *3'BCR *on der(6)
#42*	der(9)ins(9;22)(q34;q11q11), der(22)t(20;22)(q13.33;q11)	masked Ph	-	der(9)	*5'ABL *on der(9), *3'BCR *on der(20)
#43*	der(22)t(12;22)(p13;q11)ins(22;9)(q11;q34q34)	masked Ph	del(9)(q34)	der(22)	*5'ABL *deleted, *3'BCR *on der(12)
**Cryptic insertions**					
#44	ins(9;22)(q34;q11q11)	Ph^-^	del(9)(q34), del(22)(q11q12)	der(9)	*5'ABL *deleted, *3'BCR *on der(22)
#45	der(22)t(9;22)(q34;q11)ins(22;22)(q11;q11q12)	Ph^-^	-	der(22)	*5'ABL *on der(9), *3'BCR *on der(22)
#46*	der(9)ins(22;9)(q11;q34q34)t(9;11)(p22.3;p15.4)	Ph^-^	-	der(22)	*5'ABL *on der(9), *3'BCR *on der(22)
#47*	ins(9;22)(q34;q11q11), t(1;20;21)	Ph^-^	-	der(9)	*5'ABL *on der(9), *3'BCR *on der(22)
#42*	der(9)ins(9;22)(q34;q11q11), der(22)t(20;22)(q13.33;q11)	masked Ph	-	der(9)	*5'ABL *on der(9), *3'BCR *on der(20)
#43*	der(22)t(12;22)(p13;q11)ins(22;9)(q11;q34q34)	masked Ph	del(9)(q34)	der(22)	*5'ABL *deleted, *3'BCR *on der(12)
**Additional chromosomal rearrangements**					
#48	t(9;22)(q34;q11), del(11)(q14q24)	Ph^+^	del(11)(q14q24)	Ph	*5'ABL *on der(9), *3'BCR *on der(9)
#49	t(9;22)(q34;q11), t(14;15)(q32.31;q24.1)	Ph^+^	del(14)(q32), del(22)(q11)	Ph	*5'ABL *on der(9), *3'BCR *on der(9)
#50	der(9)t(9;22)(q34;q11)t(9;11)(p24.1;q12.1)	Ph^+^	-	Ph	*5'ABL *on der(9), *3'BCR *on der(9)
#46*	der(9)ins(22;9)(q11;q34q34)t(9;11)(p22.3;p15.4)	Ph^-^	-	der(22)	*5'ABL *on der(9), *3'BCR *on der(22)
#47*	ins(9;22)(q34;q11q11), t(1;20;21)	Ph^-^	-	der(9)	*5'ABL *on der(9), *3'BCR *on der(22)

Three subgroups of CML patients have been identified according to the chromosomal rearrangements detected by FISH experiments: cases showing variant t(9;22) rearrangements, patients characterized by cryptic insertions of the *ABL1 *into the *BCR *region (or vice versa), and cases bearing additional chromosomal abnormalities. Cases #1- #36 showed a Ph^+ ^chromosome together with a masked der(9) whereas a masked Ph in association with a classic der(9) was revealed in patients #37- #43. For each subgroup, the chromosomal rearrangements identified, the appearance of the der(22) chromosome, the occurrence of microdeletions next to chromosomal breakpoints, the location of the *5'BCR/3'ABL1 *and of the *3'ABL1/5'BCR *fusion genes are reported.

* indicates cases included in several cytogenetic subgroups.

#### 1. Variant t(9;22) rearrangements

**Figure 1:**
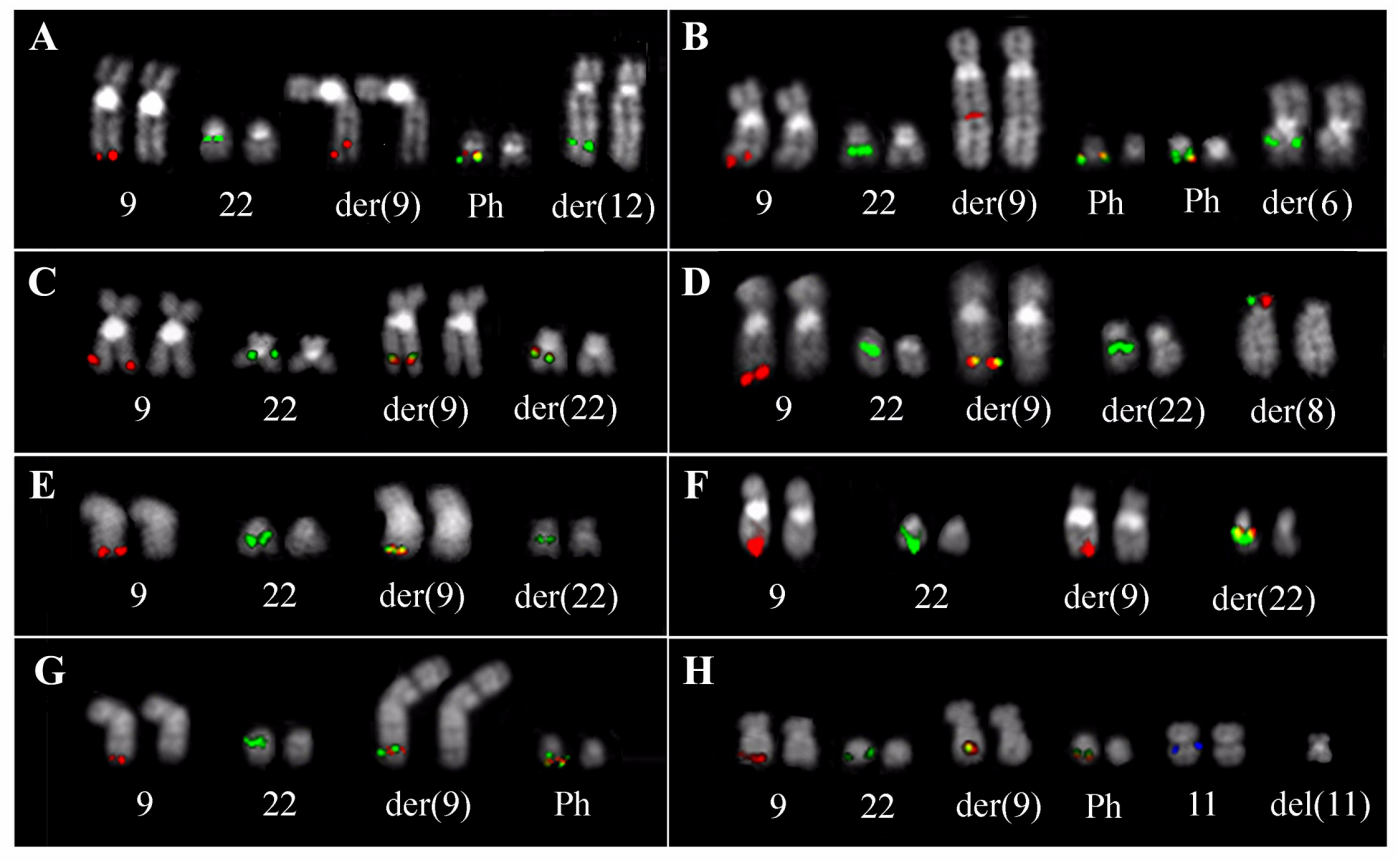
**FISH pattern observed with specific probes for the *ABL1 *and *BCR *genes on bone marrow metaphases from the analyzed CML patients**. Examples from each of the identified cytogenetic groups are shown: "masked der(9)" (A, B), "masked Ph" (C, D), "cryptic insertions" (E, F), and "concomitant rearrangements" (G, H).

Forty-three (9.5%) out of 452 CML patients showed the involvement of one (90.7%) or more chromosomes (9.3%) in addition to 9 and 22. These complex variant translocations generated a classic Ph together with a masked der(9) in 36 out of 43 cases (83.7%) and a masked Ph in association with a classic der(9) chromosome in 7 patients (16.3%) (Table 1). Cases with a masked der(9) showed the presence of additional material belonging to partner chromosomes other than chromosome 22 (Fig. 1A, B). Several chromosomes were involved in these variant translocations, with a prevalence of chromosomes 4, 6, 12, and 17 (Table 1). The *5'BCR*/*3'ABL1 *fusion gene was localized on the Ph chromosome in all these cases, whereas the *5'ABL1*/*3'BCR *gene was retained on the der(9) only in 4 (11.1%) out of 36 patients (Table 1). In the remaining 32 (88.9%) cases, the *5'ABL1*/*3'BCR *gene was not detected on der(9) due to deletions and/or 3'*BCR *transfer onto partner chromosomes (Table 1; Fig. 1A, B). Molecular cytogenetic characterization performed to verify the presence of microdeletions at the level of the rearrangements breakpoints revealed sequences loss in 18 out of the 43 (42%) cases. Among these 18 patients, 10 (55.6%) showed microdeletions of sequences belonging to the third partner chromosome, revealing a high incidence of this kind of deletions in t(9;22) variant rearrangement cases (Table 1).

Among 7 CML cases with a "masked Ph" chromosome, 3 showed the *5'BCR*/*3'ABL1 *fusion signal on 22q11, the second breakpoint on the derivative chromosome 22 mapping inside chromosome 9 sequences distal to the *ABL1 *gene (Table 1; Fig. 1C). In 3 cases the fusion gene was detected on the third partner chromosome, the second chromosome 22 breakpoint being localized centromerically to the *BCR *gene (Table 1; Fig. 1D). In patient #42 the insertion of 5' *BCR *into the *ABL1 *gene caused the *5'BCR*/*3'ABL1 *localization on der(9). The *5'ABL1*/*3'BCR *gene was detected on the der(9) in 3 cases with masked Ph, was deleted in case #39 whereas in the remaining patients the *5'ABL1 *gene was retained on the der(9) and the *3'BCR *gene was transferred onto other derivative chromosomes (Table 1). Chromosome 9 sequences loss next to the rearrangement breakpoint was observed in case #43 and an unusual loss of a region of about 400 Kb localized telomerically to the *ABL1 *gene was detected in case #41 [22] (Table 1).

#### 2. Cryptic insertions

Six (1.3%) out of the 452 CML cases showed cryptic insertions of *ABL1 *into *BCR*, or vice versa, as the cause of the *5'BCR*/*3'ABL1 *fusion gene generation (Fig. 1E, F). Four (66.7%) of these cases are indicated as "Ph negative" (Ph^-^), with chromosome 22 appearing normal without the presence of additional genomic material (Table 1). Two (33.3%) out of these 6 cases were also included in the previous group as they showed variant rearrangements generating a masked Ph (Table 1). The *5'BCR*/*3'ABL1 *gene was detected on the der(9) or on the der(22) at a ratio of 1:1 as a consequence of *5' BCR *insertion in 9q34 or *3' ABL1 *insertion in 22q11, respectively (Table 1).

#### 3. Chromosomal rearrangements concomitant to the presence of 5'BCR/3'ABL1

Conventional and molecular cytogenetic analysis showed 5 (1.1%) out of 452 CML cases bearing additional chromosomal rearrangements concomitant to the generation of the *5'BCR/3'ABL1 *fusion gene (Table 1; Fig. 1G, H). Cases #46 and #47 were also included in the previous patients group as they showed cryptic insertions; the remaining 3 CML cases carried a classic Ph chromosome.

### Bioinformatic analysis of breakpoints on other chromosomes involved in variant t(9;22) or in concomitant chromosomal rearrangements

FISH experiments with BAC clones specific for other chromosomes involved in variant or additional chromosomal rearrangements revealed a total number of 58 breakpoints. These breakpoints were mapped within a single BAC clone or in the region between two overlapping or adjacent clones (Table 2). In cases with sequences loss, two different breakpoints were defined at the level of the deleted regions boundaries.

**Table 2:** Bioinformatic analysis of the analyzed breakpoints

Cytogenetic Band	Case	Molecular Breakpoint	250 Kb interval	ALU	LINE	REF SEQ (IB)	REF SEQ (IC)	SD (IC)	SD (IB)	GC
1p34.2	2	RP11-318G20_RP11-632A13	chr1:40,158,249-40,408,249	2,27	0,77	0,71	2,16	0,67	0,21	1,06
1q32.1	1	RP11-219P13_RP11-1089F13	chr1:203,949,120-204,199,120	1,13	0,78	1,11	3,09	0,44	0,27	1,07
1q32.1	1	RP11-145I13_RP11-57I17	chr1:205,496,738-205,746,738	0,52	1,55	0,76	1,85	0,22	0,15	0,91
2q37.1	4	RP11-332L11_RP11-94I20	chr2:234,468,641-234,718,641	0,69	0,94	1,3	1,01	0	0	1,05
3p21.31	5	RP11-804H15_RP11-3B7	chr3:49,112,155-49,393,337	4,17	0,16	1,7	7,05	0	0	1,24
3p21.31	6	RP11-352L13_RP11-419G15	chr3: 48,395,535-48,645,535	2,37	0,38	1,84	9,88	0,77	0,44	1,32
3p21.31	7	RP11-316 M24	chr3:48,787,955-49,037,955	4,6	0,45	2,02	6,58	0	0	1,18
4p16.2	11	RP11-323F5_RP11-341O1	chr4:4,566,405-4,816,405	1,22	0,54	0,33	0,65	0	0	1,18
4p16.3	8	RP11-919N24	chr4:2,277,155-2,527,155	3,37	0,56	1,9	1,31	0,38	0,33	1,34
4q12	10	RP11-680 M13_RP11-167A8	hr4:58,262,438-58,512,438	0,39	1,34	0	0	0	0	0,94
4q12	10	RP11-622J1_RP11-793H8	chr4:58,796,340-59,046,340	0,5	1,28	0	0	0	0	0,95
6p12.3	12	RP3-347E1_RP11-446F17	chr6: 46,562,151-46,812,151	0,67	1,1	1,76	1,72	0	0	0,95
6p12.3	12	RP1-142O9_RP11-28H17	chr6:49,380,518-49,630,518	0,82	1,04	0,93	1,72	0	0	0,92
6p21.31	13	RP11-666K4_RP11-652G7	chr6: 36,095,599-36,345,599	1,52	0,82	1,63	4,32	0	0	1,09
6p22.1	41	RP11-635O11	chr6:27,927,951-28,177,951	1,2	0,78	0,21	4,75	1,05	0,49	0,98
6q14.1	14	RP11-1063N1_RP11-422O8	chr6:83,741,988-83,991,988	1,21	0,86	2,37	2,16	0	0	0,95
7p14.3	15	RP11-803J20_RP11-350H1	chr7:32,789,214-33,039,214	1,91	0,65	1,06	2,54	0,92	5,42	1,05
7q22.2	37	RP11-251G23	chr7:104,889,282-105,139,282	2,55	0,5	1,95	2,12	0,61	0,07	1,09
8p12	38	RP11-346L1_RP11-113G10	chr8:37,576,531-37,826,531	2,13	0,44	1,01	4,09	0	0	1,2
9p22.3	46	RP11-307K19_RP11-518K17	chr9:15,456,627-15,734,271	1,47	1,44	2,16	1,87	0	0	0,93
9p24.1	50	RP11-1084A8	chr9:6,611,140-6,861,140	3,44	0,34	1,78	2,34	0,16	0,05	1,07
9q22.31	17	RP11-412A12	chr9:93,506,167-93,756,167	1,18	0,78	2,32	0,46	0	0	1,12
10p11.22	39	RP11-241I20	chr10:32,250,540-32,500,540	2,18	0,68	0,49	0,82	0,47	0,15	1,02
11p15.4	46	RP11-120E20	chr11:3,312,588-3,573,461	1,56	0,64	0,43	1,1	40,59	18,97	1,1
11q12.1	50	RP11-624G17	chr11:56,933,830-57,183,830	2,55	0,44	1,1	3,85	0	0	1,17
11q13.1	19	RP11-665N17	chr11:64,231,470-64,481,470	2,03	0,24	1,73	6,88	0	0	1,32
11q13.1	19	RP11-821O7_RP11-755F10	chr11:65,514,670-65,764,670	1,28	1,4	2,01	2,2	0	0	1,11
11q14.2	48	RP11-185J12	chr11: 85,624,712-85,874,712	1,45	1,09	1,6	3,02	0	0	0,96
11q21	20	RP11-8N17	chr11:95,707,670-95,957,670	0,45	1,6	0,49	0,82	0	0	0,9
11q24.2	48	RP11-417F7	chr11:124,646,245-124,896,245	1,21	0,52	2	0,82	0	0	1,13
12p13.32	43	RP11-319E16	chr12:5,129,118-5,379,118	0,41	0,94	0	0	0	0	1,04
12q13.13	41	RP11-199A1_RP11-714I16	chr12:50,354,699-50,604,699	1,17	0,52	1,47	2	4,8	0,33	1,14
12q13.2	22	RP11-559I11_RP11-973D8	chr12:54,434,528-54,684,528	2,86	0,38	1,65	6,35	0,48	0,48	1,11
12q23.3	21	RP11-643D8_RP11-711H11	chr12:103,184,790-103,434,790	1,41	0,58	1,33	2,33	0	0	1,07
12q24.21	24	RP11-812F13_RP11-379F8	chr12:114,736,981-114,986,981	0,91	0,86	0,98	0,33	0	0	1,01
12q24.31	23	RP11-463O12_RP11-197N18	chr12:121,846,884-122,096,884	2,14	0,57	1,91	4,67	0	0	1,25
12q24.31	23	RP11-338K17_RP11-380L11	chr12:122,702,493-122,952,493	2,21	0,72	2,18	1,67	0	0	1,1
13q14.12	25	RP11-106H11	chr13:45,408,774-45,658,774	0,98	0,88	2,43	3,5	0	0	0,98
13q14.12	25	RP11-24B19	chr13:50,678,660-50,928,660	0,9	0,65	2,09	4,38	0	0	1,06
14q11.2	18	RP11-749G5_RP11-298I3	chr14:22,280,500-22,530,500	3,01	0,39	1,38	10,02	0	0	1,16
14q24.2	27	RP11-667E7	chr14:71,197,805-71,447,805	1,05	1,2	0,81	0,41	0	0	1,01
14q32.31	49	RP11-796G6_RP11-350L3	chr14:101,148,480-101,398,480	1	1,41	1,07	2,5	0,44	0,44	1,07
14q32.31	49	RP11-114H15_RP11-356L8	chr14:101,630,161-101,880,161	2,78	0,54	2,38	2,92	0	0	1,14
15q22.2	27	RP11-74K1	chr15:58,405,774-58,655,774	1,06	0,44	1,7	3,7	0,41	0,14	0,96
15q24.1	40	RP11-247C2	chr15:72,080,168-72,330,168	1,06	0,56	1	8,53	3,28	2,71	1,19
15q24.1	49	RP11-247C2	chr15:72,080,168-72,330,168	1,06	0,56	1	8,53	3,28	2,71	1,19
15q24.3	28	RP11-20 M10	chr15:75921863-76171863	0,88	0,47	0,82	1,48	6,78	5,58	1,2
16p11.2	29	RP11-779B17	chr16:31,765,790-32,015,790	2,15	0,56	0,39	0,28	6,11	5,91	0,93
16q24.3	30	RP11-79A1	chr16:88,199,243-88,449,243	1,92	0,21	1,86	5,1	0	0	1,21
17p13.1	30	RP11-89D11	chr17:7,395,110-7,645,110	1,66	0,64	1,56	6,66	0,29	0	1,1
17p13.3	33	RP5-1029F21	chr17:267,052-517,052	1,11	1,18	1,49	0,52	0,58	0,23	1,02
17q21.2	31	RP11-156A24	chr17:36,799,760-37,049,760	0,39	0,52	0,47	2,45	1,59	1,92	1,1
17q25.3	32	RP11-46E14	chr17:75,272,119-75,522,119	0,83	0,63	0,22	0,87	0,29	0,93	1,2
19q13.32	34	RP11-846 M4	chr19:51,871,627-52,121,627	1,41	0,64	0,93	1,77	0	0	1,03
20q13.33	42	RP4-591C20_RP11-266K16	chr20:62,058,835-62,308,835	0,95	0,29	1,47	4,29	1,87	0,91	1,27
21q22.13	35	RP11-315B15_RP11-777J19	chr21:37,536,287-37,809,765	2,2	0,89	1,77	2,67	0	0	0,98
21q22.13	35	RP11-105O24_RP11-1021I19	chr21:37,747,110-37,997,110	0,98	1,02	1,57	2,67	0	0	1,04
Xq13.1	36	RP11-69L22_RP11-237F13	chrX:68,788,268-69,038,268	0,66	1,58	3,32	1,95	0	0	0,97

The 250 Kb size intervals covering the molecular breakpoints were analyzed for the presence of *Alu*, LINE, RefSeq Genes, SDs, and GC. The reported values were normalized to the mean value for each chromosome. In cases characterized by sequences deletions or the involvement of several chromosomes, more than one molecular breakpoints was identified.

**Figure 2:**
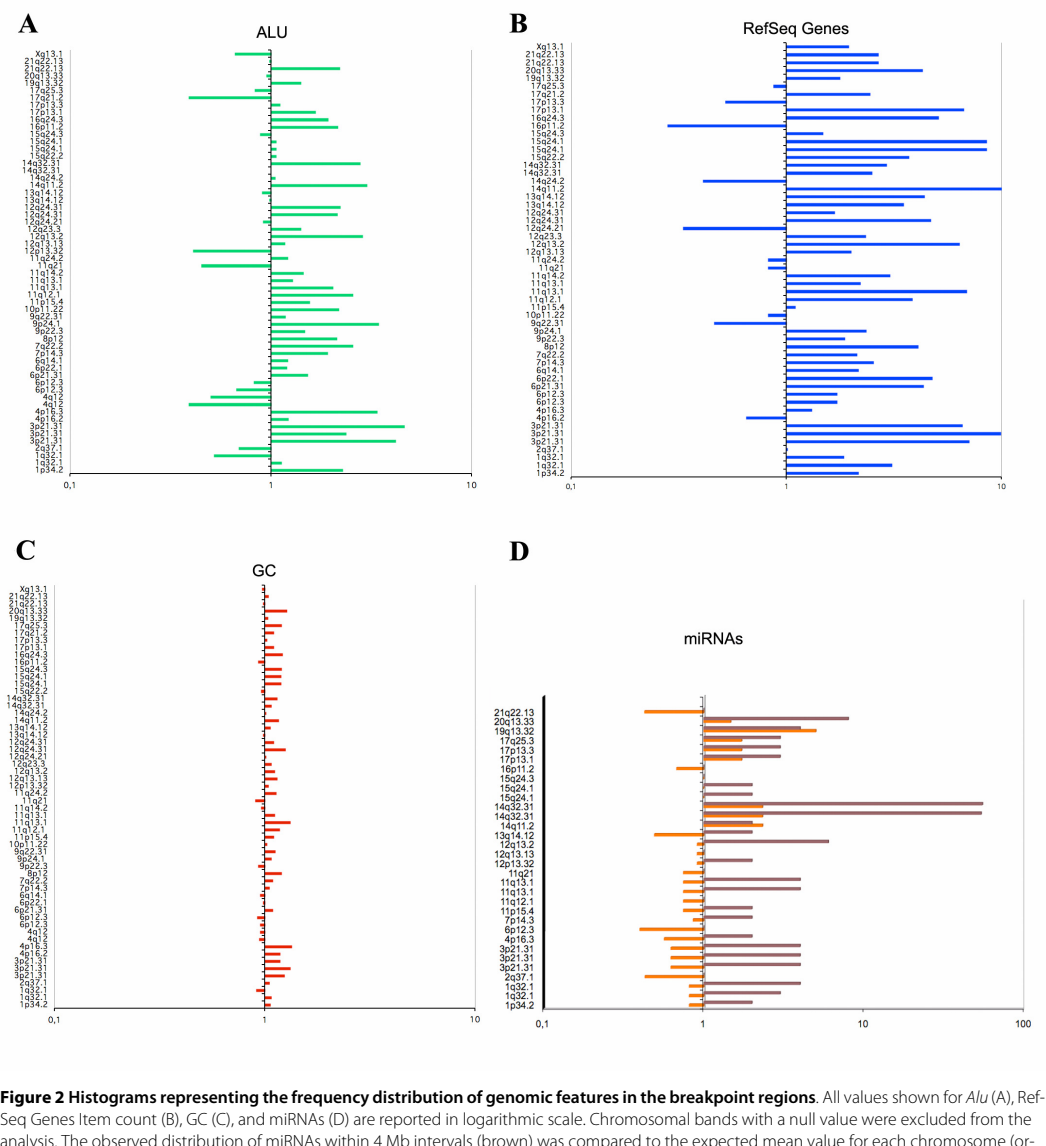
**Histograms representing the frequency distribution of genomic features in the breakpoint regions**. All values shown for *Alu *(A), RefSeq Genes Item count (B), GC (C), and miRNAs (D) are reported in logarithmic scale. Chromosomal bands with a null value were excluded from the analysis. The observed distribution of miRNAs within 4 Mb intervals (brown) was compared to the expected mean value for each chromosome (orange).

Interestingly, the majority of breakpoints on chromosomes involved in variant or additional chromosomal rearrangements showed a high frequency of *Alu *repeats (Table 2; Fig. 2A). In fact, 41 out of 58 (71%) breakpoints showed an *Alu *content of more than one whereas the remaining 17 out of 58 (29%) had a content of less than one. Instead, the LINE content was lower than one in 44 out of 58 (76%) breakpoints (Table 2). Thirty-five out of 41 breakpoints (85%) with *Alu *>1 showed a LINE amount < 1 (Table 2).

Most of the analyzed breakpoints map within gene-rich regions as a RefSeq Genes Item count of more than one was observed in 45 out of 58 (78%) breakpoints (Table 2; Fig. 2B). Moreover, 40 out of 58 (69%) breakpoints showed a RefSeq Genes Item bases value of more than 1 (Table 2). It is worthy of note that 34 out of 41 bp (83%) with *Alu *>1 showed a RefSeq Genes Item count >1 (Table 2). The number of known genes localized at breakpoints and their function as oncogenes and/or TSGs are reported in Table 3.

**Table 3:** Genes with known function identified in breakpoint regions

Cytogenetic Band	Case	Molecular Breakpoint	Breakpoint region	Deleted region	Number of known genes	TSG/Oncogenes
1p34.2	2	RP11-318G20_RP11-632A13	chr1:40,270,704-40,309,712		1	
1q32.1	1	RP11-1089F13_RP11-145I13		chr1:203,989,316-205,431,690	26	*IKBKE, RASSF5, IL10, IL24*
2q37.1	4	RP11-332L11_RP11-94I20	chr2:234,593,254-234,594,028		0	
3p21.31	5	RP11-804H15_RP11-3B7	chr3:49,112,155-49,222,551		8	
3p21.31	6	RP11-352L13_RP11-419G15	chr3:48,468,265-48,572,806		4	
3p21.31	7	RP11-316 M24	chr3:48,859,939-48,965,972		4	
4p16.2	11	4ptel_RP11-341O1		chr4:0-4,673,371	68	*GAK*
4p16.3	8	RP11-919N24	chr4:2,317,528-2,486,782		2	
4q12	10	RP11-167A8_RP11-622J1		chr4:58,389,737-58,916,984	0	
6p12.3	12	RP11-446F17_RP1-142O9		chr6: 46,562,151-46,812,151	14	
6p21.31	13	RP11-666K4_RP11-652G7	chr6:36,174,067-36,267,130	chr6:46,715,042-49,457,682	2	
6p22.1	41	RP11-635O11	chr6:27,960,335-28,145,568		5	
6q14.1	14	RP11-1063N1_RP11-422O8	chr6:83,875,190-83,886,368		1	
7p14.3	15	RP11-803J20_RP11-350H1	chr7:32,469,044-33,359,385		10	
7q22.2	37	RP11-251G23	chr7:104,957,075-105,071,488		3	*RINT1*
8p12	38	RP11-346L1_RP11-113G10	chr8:37,687,186-37,715,876		1	
9p22.3	46	RP11-307K19_RP11-518K17	chr9:15,455,865-15,456,627		1	
9p24.1	50	RP11-1084A8	chr9:6,611,140-6,861,140		2	*KDM4C*
9q22.31	17	RP11-412A12	chr9:93,553,640-93,708,694		1	
10p11.22	39	RP11-241I20	chr10:32,303,829-32,447,252		1	
11p15.4	46	RP11-120E20	chr11:3,573,461-3,758,006		5	*ART1, NUP98*
11q12.1	50	RP11-624G17	chr11:56,953,550-57,164,109		10	
11q13.1	19	RP11-665N17_RP11-821O7		chr11:64,481,470-65,571,636	57	*FAU, SYVN1, RELA, CFL1, CST6*
11q14.2	48	RP11-185J12_RP11-417F7		chr11: 85,874,712-124,646,245	297	*TSG11, CADM1, FAT3, MLL, ATM, YAP1, ZBTB16, THY1, SDHD, ARHGEF12, BTG4, PPP2R1B, NOX4, POU2F3, TBRG1, TAGLN, CBL*
11q21	20	RP11-8N17	chr11:95,755,457-95,909,884		2	
12p13.32	43	RP11-319E16	chr12:5,163,930-5,344,301		0	
12q13.13	41	RP11-199A1_RP11-714I16	chr12:50,423,209-50,425,214		1	
12q13.2	22	RP11-559I11_RP11-973D8	chr12:54,583,954-54,618,511		2	
12q23.3	21	RP11-643D8_RP11-711H11	chr12:103,271,502-103,364,761		0	
12q24.21	24	RP11-812F13_RP11-379F8	chr12:114,705,782-115,018,181		1	
12q24.31	23	RP11-197N18_RP11-338K17		chr12:121,978,125-122,877,008	19	
13q14.12	25	RP11-106H11_RP11-24B19		chr13:45,658,774-50,678,660	33	*RB1, ARL11, TRIM13, FAM10A4*
14q11.2	18	RP11-749G5_RP11-298I3	chr14:22,375,744-22,435,254		3	
14q24.2	27	RP11-667E7	chr14:71,230,541-71,415,068		1	
14q32.31	49	RP11-350L3_RP11-114H15		chr14:101,273,490-101,716,645		*HSP90AA1*
15q22.2	27	RP11-74K1	chr15:58,530,516-58,531,032		1	
15q24.1	40	RP11-247C2	chr15:72,158,366-72,251,969		3	
15q24.1	49	RP11-247C2	chr15:72,158,366-72,251,969		3	
15q24.3	28	RP11-20 M10	chr15:75,965,658-76,128,067		3	
16p11.2	29	RP11-779B17	chr16:31,786,062-31,995,517		1	
16q24.3	30	RP11-79A1	chr16:88,125,792-88,522,693		9	
17p13.1	30	RP11-89D11	chr17:7,436,436-7,603,767		18	*TP53*
17p13.3	33	RP5-1029F21	chr17:343,377-440,727		1	*VPS53*
17q21.2	31	RP11-156A24	chr17:36,841,610-37,007,910		9	
17q25.3	32	RP11-46E14	chr17:75,316,874-75,477,363		4	
19q13.32	34	RP11-846 M4	chr19:51,889,293-52,103,962		6	
20q13.33	42	RP4-591C20_RP11-266K16	chr20:62,100,354-62,267,316		9	
21q22.13	35	RP11-777J19_RP11-105O24		chr21:37,641,259-37,872,927	1	
Xq13.1	36	RP11-69L22_RP11-237F13	chrX:68,857,148-68,969,387		1	

The number of the genes with known function mapping in the breakpoint regions or located inside the deleted regions was reported for each case. Known oncogenes and TSGs have been identified according to the NCBI.

In the search for SDs, 49 out of 58 (84%) and 51 out of 58 (88%) breakpoints revealed SDs Item count and SDs Item bases of less than one, respectively (Table 2). In cases showing the presence of SDs within breakpoint regions no specific association with chromosomes 9 and 22 was detected, as the duplicated elements recognized several chromosomal regions.

Finally, a GC content >1 was detected in 43 out of 58 (74%) breakpoints (Table 2; Fig. 2C). A GC content of more than one was identified in 34 out of 41 (83%) breakpoints with *Alu *>1 and in 34 out of 45 (76%) with a RefSeq Genes Item count >1 (Table 2).

The search for miRNAs revealed a different density from the expected value in 32 out of 58 (55%) breakpoint regions (Fig. 2D). In detail, in 30 (94%) and 2 out of 32 (6%) breakpoints a higher or lower number of miRNA than the expected value was identified, respectively (Fig. 2D). In the remaining 26 out of 58 (45%) breakpoints no miRNA was revealed in the 4 Mb analyzed intervals. It is noteworthy that in case #49 with an additional t(14;15)(q32;q24) a miRNA cluster of 54 elements was revealed in the 14q32 breakpoint region. In this patient a microdeletion of about 450 Kb was detected on 14q32, resulting in the loss of almost the entire miRNA cluster. The list of miRNAs found at the breakpoints is reported in Table 4; in addition to the 14q32 miRNA cluster a total number of 63 known miRNA was identified, 8 (13%) of which show involvement in hematological malignancies. Moreover, querying the miRGen database (the intersection data from the miRanda, PicTar, and TargetScan programs) allowed the identification of the predicted target genes in 19 out of 63 (30%) analyzed miRNAs (see Additional File 1). Among the identified target genes, several play a role as oncogenes or TSGs (see Additional File 1). Noteworthy, some miRNAs share the same target oncogenes or TSGs; for example, *PPM1D *(protein phosphatase, Mg2+/Mn2+ dependent, 1D) and *AKT3 *(v-akt murine thymoma viral oncogene homolog 3) genes are the most frequent miRNAs targets (see Additional File 1).

**Table 4:** Known miRNA mapped at the breakpoint regions.

Cytogenetic Band	miRNA	miRNA position	Hematologic Malignancies involvement
1p34.2	mir-30e	chr1:40,992,614-40,992,705	no
	mir-30c-1	chr1:40,995,543-40,995,631	no
1q32.1	mir-135b	chr1:203,684,053-203,684,149	no
	mir-29c	chr1:206,041,820-206,041,907	CLL^36,37^, ALL^37^
	mir-29b-2	chr1:206,042,411-206,042,491	AML^38^, ALL^38^
	mir-205	chr1:207,672,101-207,672,210	no
2q37.1	mir-562	chr2:232,745,607-232,745,701	no
3p21.31	mir-1226	chr3:47,866,049-47,866,123	no
	mir-425	chr3:49,032,585-49,032,671	no
	mir-191	chr3:49,032,805-49,033,396	AML^39^, ALL^40^
	mir-566	chr3:50,185,763-50,185,856	no
4p16.3	mir-943	chr4:1,957,909-1,958,002	no
	mir-571	chr4:333,946-334,041	no
6p12.3	mir-586	chr6:45,273,389-45,273,485	no
7p14.3	mir-550-2	chr7:32,739,118-32,739,214	no
	mir-548n	chr7:34,946,897-34,946,971	no
11p15.4	mir-675	chr11:1,974,565-1,974,637	no
	mir-483	chr11:2,111,940-2,112,015	no
11q12.1	mir-130a	chr11:57,164,997-57,165,585	no
11q13.1	mir-1237	chr11:63,892,650-63,892,751	no
	mir-192	chr11:64,414,935-64,415,544	no
	mir-194-2	chr11:64,415,153-64,415,737	no
	mir-612	chr11:64,968,505-64,968,604	no
11q21	mir-548l	chr11:93,839,309-93,839,394	no
12p13.32	mir-200c	chr12:6,942,873-6,943,440	no
	mir-141	chr12:6,943,271-6,943,865	no
12q13.13	mir-1293	chr12:48,914,192-48,914,262	no
12q13.2	mir-196a-2	chr12:52,671,789-52,671,898	no
	mir-615	chr12:52,714,001-52,714,096	no
	mir-148b	chr12:53,017,267-53,017,365	no
	mir-1228	chr12:55,874,554-55,874,626	no
	mir-616	chr12:56,199,213-56,199,309	no
	mir-26a-2	chr12:56,504,409-56,504,992	no
13q14.12	mir-16-1	chr13:49,521,110-49,521,198	CLL^41^, MDS^42^
	mir-15a	chr13:49,521,006-49,521,588	CLL^43^, MM^44^
14q11.2	mir-208a	chr14:22,927,645-22,927,715	no
	mir-208b	chr14:22,957,036-22,957,112	no
14q32.31	miRNA cluster 1	chr14:99,645,745--101,096,512	AML^45^, B-cell malignancies^46^
14q32.31	miRNA cluster 2	chr14:99,645,745-103,653,604	AML^45^, B-cell malignancies^46^
15q24.1	mir-630	chr15:70,666,612-70,666,708	no
	mir-631	chr15:73,433,005-73,433,079	B cell lymphomas^47^
15q24.3	mir-184	chr15:77,289,185-77,289,268	no
16p11.2	mir-1826	chr16:33,873,009-33,873,093	no
17p13.1	mir-195	chr17:6,861,408-6,861,994	CLL^37^, ALL^37^
	mir-497	chr17:6,861,954-6,862,065	no
	mir-324	chr17:7,067,340-7,067,422	no
17p13.3	mir-22	chr17:1,563,947-1,564,031	no
	mir-132	chr17:1,899,952-1,900,052	T-cell leukemia^48^
	mir-212	chr17:1,900,315-1,900,424	no
17q25.3	mir-657	chr17:76,713,671-76,713,768	no
	mir-338	chr17:76,714,278-76,714,344	no
	mir-1250	chr17:76,721,591-76,721,703	no
19q13.32	mir-330	chr19:50,834,092-50,834,185	no
	mir-642	chr19:50,870,026-50,870,122	no
	mir-769	chr19:51,214,030-51,214,147	no
	mir-220c	chr19:53,755,341-53,755,423	no
20q13.33	mir-1-1	chr20:60,561,708-60,562,278	no
	mir-133a-2	chr20:60,572,314-60,572,915	no
	mir-124-3	chr20:61,280,297-61,280,383	no
	mir-941-1	chr20:62,021,238-62,021,326	no
	mir-941-2	chr20:62,021,545-62,021,633	no
	mir-941-3	chr20:62,021,657-62,021,745	no
	mir-1914	chr20:62,043,262-62,043,341	no
	mir-647	chr20:62,044,428-62,044,523	no
21q22.13	mir-802	chr21:36,014,883-36,014,976	no

For each cytogenetic band identified in this study are reported known miRNAs, the mapping position derived from the UCSC database querying, and their implication in hematological malignancies according to literature data.

### Identification of cytogenetic hotspots

Our study revealed 46 cytogenetic breakpoints on other chromosomes involved in variant t(9;22) rearrangements (see Additional File 2). The assessment of the O/E ratio for each breakpoint allowed us to identify 24 hotspots, 12 of which have been previously described in literature [26] (see Additional File 2). Notably, 4 out of 12 new hotspots showed a ratio >2 involving the chromosomal bands 4q12, 9p11, 11q21 and 21q22 (see Additional File 2).

To investigate the breakpoints distribution in the genome, a review of literature data about variant t(9;22) following the study by Fisher *et al*. was carried out [4,30-33]. In total, 60 new hotspots were identified, 18 of which have already been reported [26]. However, 10 previously published hotspots were not supported by our literature review (see Additional File 2). Among the 60 new hotspots, 27 showed an O/E ratio > 2.

### Treatment response

Data on the response to treatment in the analyzed CML patients were only available for about 50% of the cases; a summary is shown in Table 5. All the cases evaluable for the response to interferon-α therapy were non responders whereas 11 out of 17 (65%) cases treated with imatinib achieved cytogenetic response. Among patients resistant to imatinib, 3 (75%) treated with dasatinib achieved CCyR.

**Table 5:** Response to treatment of 50 CML patients included in the study

Case	Hydroxyurea	Interferon-α	Imatinib	Nilotinib	Dasatinib
#1	*NE*	*NE*	*NR*	*NR*	*CCyR*
#2	*NE*	*NE*	*CCyR*	*NE*	*NE*
#3	*NA*	*NA*	*NA*	*NA*	*NA*
#4	*NA*	*NA*	*NA*	*NA*	*NA*
#5	*NE*	*NE*	*CCyR*	*NE*	*NE*
#6	*NE*	*NE*	*NR*	*NE*	*NE*
#7	*NA*	*NA*	*NA*	*NA*	*NA*
#8	*NA*	*NA*	*NA*	*NA*	*NA*
#9	*NA*	*NA*	*NA*	*NA*	*NA*
#10	*NA*	*NA*	*NA*	*NA*	*NA*
#11	*NE*	*NR*	*NE*	*NE*	*NE*
#12	NR*	*NE*	*NE*	*NE*	*NE*
#13	*NE*	*NE*	*CCyR*	*NE*	*NE*
#14	*NA*	*NA*	*NA*	*NA*	*NA*
#15	*NA*	*NA*	*NA*	*NA*	*NA*
#16	*NA*	*NA*	*NA*	*NA*	*NA*
#17	*NE*	*NE*	*CCyR*	*NE*	*NE*
#18	*NA*	*NA*	*NA*	*NA*	*NA*
#19	*NE*	*NE*	*CCyR*	*NE*	*NE*
#20	*NA*	*NA*	*NA*	*NA*	*NA*
#21	*NE*	*NE*	*CCyR*	*NE*	*NE*
#22	*NA*	*NA*	*NA*	*NA*	*NA*
#23	*NA*	*NA*	*NA*	*NA*	*NA*
#24	*NA*	*NA*	*NA*	*NA*	*NA*
#25	*NE*	*NR*	*NR*	*NE*	*CyCR*
#26	*NE*	*NR*	*CCYR*	*NE*	*NE*
#27	*NA*	*NA*	*NA*	*NA*	*NA*
#28	*NA*	*NA*	*NA*	*NA*	*NA*
#29	*NA*	*NA*	*NA*	*NA*	*NA*
#30	*NE*	*NE*	*CCyR*	*NE*	*NE*
#31	*NE*	*NE*	*NR*	*NE*	*NE*
#32	*NA*	*NA*	*NA*	*NA*	*NA*
#33	*NA*	*NA*	*NR*	*NE*	*NR*
#34	*NA*	*NA*	*NA*	*NA*	*NA*
#35	*NE*	*NE*	*CCyR*	*NE*	*NE*
#36	*NA*	*NA*	*NA*	*NA*	*NA*
#37	NR*	*NE*	*NE*	*NE*	*NE*
#38	*NE*	*NR*	*NE*	*NE*	*NE*
#39	*NA*	*NA*	*NA*	*NA*	*NA*
#40	*NE*	*NR*	*NE*	*NE*	*NE*
#41	*NE*	*NR*	*PCyR*	*NE*	*NE*
#42	*NA*	*NA*	*NA*	*NA*	*NA*
#43	*NE*	*NR*	*CCyR*	*NE*	*NE*
#44	*NA*	*NA*	*NA*	*NA*	*NA*
#45	*NA*	*NA*	*NA*	*NA*	*NA*
#46	*NE*	*CHR*	*NE*	*NE*	*NE*
#47	*NA*	*NA*	*NA*	*NA*	*NA*
#48	*NE*	*NR*	*NE*	*NE*	*NE*
#49	*NE*	*NR*	*NR*	*NE*	*CCyR*
#50	*NA*	*NA*	*NA*	*NA*	*NA*

NE = Not Evaluable; NA = Not Available; NR = Non Responder; CCyR = Complete Cytogenetic Response; CHR = Complete Hematologic Response; PcyR = Partial Cytogenetic Response; * = patient who died in the pre imatinib era, of blast crisis.

## Discussion

Literature data indicate that breakpoints on additional chromosomes involved in CML cases with variant t(9;22) are not distributed randomly in the genome but show hotspots [26]. Several genomic features such as the density of CpG islands, genes, *Alu *repeats, recombination events, openness of the chromatin structure and transcription activity have been correlated to the occurrence of breakpoints in variant t(9;22) cases [26,33,34].

In this study, we have performed for the first time a precise molecular cytogenetic characterization of breakpoints involved in variant t(9;22) or in additional rearrangements, in 50 CML cases. To identify genomic elements with a role in the occurrence of chromosomal translocations, bioinformatic analysis was carried out to investigate the distribution and density of several genomic features, such as *Alu*, LINE, GC, SDs, miRNAs, and genes at breakpoint regions. To date, according to the miRBase database http://www.mirbase.org[35] the total number of known miRNAs is very low (about 720) as compared to the human genome size (3.1 × 10^9 ^bp). In this study the miRNAs density within the 4 Mb analyzed intervals resulted higher than the expected value in 32 out of 58 (55%) breakpoint regions. These findings suggest a potential role for miRNAs in the pathogenesis of CML cases with variant or additional chromosomal rearrangements. Few miRNAs located at breakpoint regions have previously been described in several hematological malignancies [36-48]. However, none of them was involved in CML. It is worth noting the presence of the miRNA cluster next to the breakpoint region in 14q32 (case #49). miRNAs in this region are organized in an imprinted domain regulated by a differentially methylated region located upstream of the miRNA cluster. It has been reported that these miRNAs act as tumor suppressor genes and that changes in their methylation status could promote tumor development [49]. Querying of miRGen and NCBI databases showed the involvement of interesting target oncogenes or TSGs implicated in a wide variety of biological processes including cell proliferation, differentiation, apoptosis, and tumorigenesis.

Increasing evidence shows a high density of interspersed repetitive elements, such as *Alu *and LINE, at some chromosomal translocation breakpoints, suggesting a mediator role of some recurrent rearrangements in tumors [50]. Because a much higher density of *Alu *repeats has been observed in the DNA sequences flanking the *ABL1 *and *BCR *genes, it has been hypothesized that *Alu *elements provide hotspots for non allelic homologous recombination and mediate chromosomal translocation in CML [34,50]. Our data, supported by bioinformatic evidence, suggest that the high density of *Alu *repeats could increase the propensity to undergo rearrangements also of other chromosomes involved in variant t(9;22). In our CML series, a high *Alu *density was detected in 71% of the analyzed breakpoints. Moreover, a rich content of *Alu *repeats was revealed also on breakpoint regions identified in chromosomal rearrangements concomitant to the t(9;22).

Literature data revealed a preferential breakpoints distribution in CML cases with variant t(9;22) within the CG-richest regions of the genome corresponding to the G-light banding karyotype [26,33]. Our data confirmed this association, as 83% of the identified cytogenetic breakpoints mapped inside G-light bands. Moreover, we report the first bioinformatic evidence of the association between GC-content and breaks in cases with variant t(9;22), as 73% of the molecular breakpoints showed a GC content >1. In addition, these data showed that CG richness was related to other genomic features such as *Alu *content and a greater gene density than the mean expected value.

The search for SDs revealed a low density in the majority of the analyzed breakpoints, without showing any specific association with chromosomes 9 and 22 regions, unlike what has been reported about the occurrence of the t(9;22) in CML [51].

Moreover, our study provided an outline of the frequency and molecular features of the most relevant cytogenetic groups identified in a very large series of CML patients at diagnosis. Three-way translocations were the most frequent among variant t(9;22) rearrangements, chromosomes 4, 6, 12, and 17 being common partners. However, no cytogenetic breakpoints clustering was revealed when the same partner chromosome was rearranged, except for the 3p21 band, that was involved in 3 CML cases with variant t(9;22).

As to the mechanisms involved in the formation of the variant t(9;22) rearrangements, our data indicated that the most probable mechanism, identified in cases with a "masked der(9)" chromosome, is a single event consisting of multiple simultaneous breaks and rejoins (one-step model). In fact, splitting of the *5'ABL1*/*3'BCR *fusion gene signal was observed in the majority (27 out of 36, 75%) of analyzed cases. A two-step mechanism was hypothesized in about 11% of cases bearing a "masked der(9)" chromosome; the permanence of the *5'ABL1*/*3'BCR *gene on the der(9) suggests that a second break occurred inside the chromosome 22 sequence telomeric to the *BCR *gene. On the contrary, in 71.4% of cases (#37 - #41) with a "masked Ph" chromosome a second break located proximally to *BCR *or distally to *ABL1 *was identified, suggesting the occurrence of a two-step mechanism in the majority of the CML patients included in this group.

In our study, FISH 'walking' with BAC/PAC contigs belonging to the chromosomes 9 and 22 next to the t(9;22) breakpoint regions allowed us to assess the frequency of deletions in three main cytogenetic subgroups of CML patients and the size of these microdeletions. Confirming the deletion frequency reported in literature [12], 12 out of the 36 (33%) cases with a "masked der(9)" chromosome showed chromosome 9 and/or 22 sequences loss. Moreover, in about 55% of these patients we found extensive genomic deletions on the third chromosome, in addition to deletions on der(9). Chromosome 9 sequences deletions were detected in 3 out of 6 (50%) cases with a masked Ph (#39, #41, and #43) and in 1 out of 4 (25%) Ph^- ^cases (#44). These frequencies are higher than the value recently reported in literature [18].

The biological significance and the prognostic impact of the cytogenetic molecular heterogeneity occurring in the generation of the *5'BCR/3'ABL1 *fusion gene remain to be clarified. However, the bioinformatic analysis performed in this study on a large number of breakpoints in CML cases with variant t(9;22) or additional chromosomal alterations revealed that the rearranged regions are characterized by an elevated content of miRNAs, *Alu *repeats, GC and known genes.

In conclusion, this genomic analysis of breakpoint regions provides clues to a better understanding of the pathogenetic mechanisms that underlie CML onset. Further analyses will be needed to demonstrate the functional meaning of these genomic features.

## List of abbreviations

(CP-CML): Chronic Myeloid Leukemia; (FISH): Fluorescence In Situ Hybridization; (CML): Chronic myeloid leukemia; (Ph): Philadelphia chromosome; (SDs): Segmental Duplications; (GTG): Giemsa-Trypsin-Giemsa; (HAL): Haploid Autosomal Length; (E): Number of breaks expected; (O): Number of breaks observed; (BAC): Bacterial artificial chromosome; (PAC): Phage P1-derived artificial chromosome; (UCSC): University of California Santa Cruz; (Ph^-^): Ph negative.

## Competing interests

The authors declare that they have no competing interests.

## Authors' contributions

FA, LA, and AZ were involved in the design and execution of the experiments, wrote the manuscript and contributed to the overall experimental design. NC conducted most of the FISH experiments. PC and LV performed conventional cytogenetic analysis; ARR contributed to clinical data collection. VL, MR and GS participated in the design of the study and supervised the manuscript preparation. All authors have read and approved the final manuscript.

## Supplementary Material

Additional file 1 **Oncogenes and TSGs regulated by the analyzed miRNAs**. The number of the predicted target genes for each analyzed miRNA was reported according to the miRGen database. Target genes with a role as oncogenes or TSGs were identified by querying the NCBI database. Click here for file

Additional file 2 **Variant t(9;22) Breakpoints**. The chromosomal bands involved in variant t(9;22) are shown according to our study and recent large series of CML patients reported in literature. The number of total breaks observed in each band is shown, together with the observed/expected (O/E) ratio. In bold are indicated cytogenetic hotspots, O/E ratio being > 1. The symbol * represents hotspots previously reported [26]. Click here for file
